# *Weizmannia coagulans* BC99 Attenuates Oxidative Stress Induced by Acute Alcoholic Liver Injury via Nrf2/SKN-1 Pathway and Liver Metabolism Regulation

**DOI:** 10.3390/antiox14010117

**Published:** 2025-01-20

**Authors:** Ying Wu, Cheng Li, Yinyin Gao, Jie Zhang, Yao Dong, Lina Zhao, Yuwan Li, Shaobin Gu

**Affiliations:** 1 College of Food and Bioengineering, Henan University of Science and Technology, Luoyang 471000, China; yingwu@haust.edu.cn (Y.W.); lc15515735845@163.com (C.L.); zhaolina@haust.edu.cn (L.Z.); liyuwan91@163.com (Y.L.); 2 Henan Engineering Research Center of Food Microbiology, Luoyang 471000, China; 3 National Demonstration Center for Experimental Food Processing and Safety Education, Luoyang 471000, China; 4 Germline Stem Cells and Microenvironment Lab, College of Animal Science and Technology, Nanjing Agricultural University, Nanjing 210095, China; yao.dong@stu.njau.edu.cn

**Keywords:** *Weizmannia coagulans*, acute alcoholic liver injury, oxidative stress, nuclear factor E2-related factor 2 (Nrf2), *Caenorhabditis elegans*

## Abstract

Acute alcoholic liver injury (AALI) remains a significant global health concern, primarily driven by oxidative stress. This study investigated the protective mechanisms of *Weizmannia coagulans* BC99 against alcohol-induced oxidative stress using a dual model in rats and Caenorhabditis elegans. In rats, excessive alcohol was predominantly metabolized via the CYP2E1 pathway, leading to severe oxidative stress. However, intervention with BC99 suppressed CYP2E1 expression and enhanced antioxidant enzyme activities through the Nrf2/SKN-1 pathway, thereby alleviating oxidative stress. Additionally, BC99 treatment elevated glutamate and aspartate levels while reducing glycerate and glucose, which collectively increased glutathione levels and mitigated oxidative stress triggered by glucose metabolism disorders. In C. elegans, BC99 reduced excessive ROS by upregulating *Nrf2*/*skn-1*, *daf-16*, and their downstream antioxidant genes, consequently alleviating the biotoxicity associated with alcohol-induced oxidative damage. The protective effects of BC99 were markedly diminished in the *skn-1* mutant (GR2245) and *daf-16* mutant (CF1038), further confirming the pivotal roles of SKN-1 and DAF-16 pathways in BC99-mediated antioxidant protection. Taken together, these findings reveal that BC99 mitigates alcohol-induced oxidative stress by activating the Nrf2/SKN-1 pathway and regulating liver metabolites to eliminate excess ROS, thereby providing a theoretical basis for the application of probiotics in preventing acute alcoholic liver injury.

## 1. Introduction

Acute alcoholic liver injury (AALI) is a hepatic disorder caused by excessive alcohol consumption over a short period [1]. According to the World Health Organization (WHO), approximately 3 million deaths worldwide are attributed to harmful drinking annually, accounting for 5.3% of all deaths [2]. The risk of mortality among AALI patients is nearly five times higher than that of the general population [3]. In its early stages, AALI typically manifests as alcoholic fatty liver disease, which, if left untreated, can progress to alcoholic hepatitis, liver fibrosis, cirrhosis, or even hepatocellular carcinoma [4]. Currently, the primary interventions for AALI involve a combination of alcohol abstinence and pharmacotherapy, with commonly used drugs including corticosteroids, reduced glutathione, and trimethoprim [5]. While these therapies provide certain benefits, they are often associated with adverse reactions and potential toxicity.

AALI pathogenesis involves multiple factors, including abnormal alcohol metabolism, oxidative stress, inflammatory responses, and disruptions in intestinal homeostasis caused by excessive alcohol intake [6,7]. Among these, oxidative stress is considered a critical driver of alcoholic liver injury [8]. Excessive alcohol consumption overwhelms the liver’s detoxification capacity, leading to acetaldehyde accumulation in the blood and liver [9]. Concurrently, ethanol activates the microsomal ethanol oxidizing system (MEOS), generating large quantities of reactive oxygen species (ROS) [10]. Ethanol metabolism also produces oxidative byproducts such as acetaldehyde, free radicals, and malondialdehyde (MDA). MDA can react with neighboring proteins to form MDA–acetaldehyde adducts, which exacerbate inflammatory responses by upregulating cytokines. Therefore, the development of natural hepatoprotective agents with potent antioxidant properties and minimal side effects is crucial for the prevention and treatment of AALI.

Probiotics are active microorganisms, and when administered in a sufficient amount, they will have beneficial effects on the host [11]. Studies have shown that probiotics can reduce the level of oxidative stress markers in the body [12] and significantly increase the activity of serum antioxidant enzymes [13]. Research has proven that *Lactobacillus fermentum* can improve ethanol-induced liver injury by scavenging reactive oxygen species (ROS) and regulating the expression of nitric oxide synthase (iNOS) and heat shock proteins [14]. Tian et al. [15] also confirmed that *Lactobacillus rhamnosus* CCFM1107 mainly removes free radicals in vivo, reduces oxidative stress and restores the balance of the intestinal flora, achieving the goal of preventing and treating alcohol-induced liver injury. However, the number of probiotics that alleviate alcohol damage is low, and further research is needed to develop potential probiotics.

*W. coagulans* is a Gram-positive, spore-forming bacterium with superior resistance to gastric acid, bile salts, heat, and oxygen compared to other probiotics [16]. In vitro studies have shown that *W. coagulans* effectively scavenges 1,1-diphenyl-2-pyridazinyl (DPPH) radicals, hydroxyl radicals, and superoxide anion radicals [17]. Furthermore, *W. coagulans* has been shown to mitigate oxidative stress in vivo by scavenging free radicals, enhancing antioxidant enzyme activity, and reducing lipid peroxidation in cell membranes [18,19]. These findings highlight the potential of *W. coagulans* as a natural hepatoprotective agent against AALI. To further elucidate its therapeutic potential and mechanisms, this study utilized a dual model comprising rats and *Caenorhabditis elegans* in combination with metabolomics analyses to investigate the effects of *W. coagulans* BC99 on alcohol-induced oxidative stress.

## 2. Materials and Methods

### 2.1. Strains and Preparation of Bacterial Suspensions

The strain *W. coagulans BC99* (hereafter referred to as BC99) was provided by Wecare Probiotics Co., Ltd. (Suzhou, China). The bacterial powder contained a concentration of 1 × 10^11^ CFU/g. The required doses of the bacterial suspension were prepared by diluting the powder with sterile saline. The prepared suspensions were stored at 4 °C until use.

### 2.2. Animal and Experimental Design

Fifty SPF-grade male Sprague-Dawley (SD) rats, aged 4–6 weeks, were obtained from SPF Biotechnology Co., Ltd. (Beijing, China). The rats were randomly divided into the control group (NC), model group (AD), and BC99 low-dose group (AL), medium-dose group (AM), and high-dose group (AH), with 10 rats in each group. Red star Erguotou with an alcohol content of 53 was purchased from Beijing Red Star Co., Ltd. (Beijing, China). The rat maintenance feed was purchased from SPF Biotechnology Co., Ltd. The animal room maintained a temperature of 21–25 °C, humidity of 48–55%, and a 12 h light/dark cycle.

**Figure 1:**
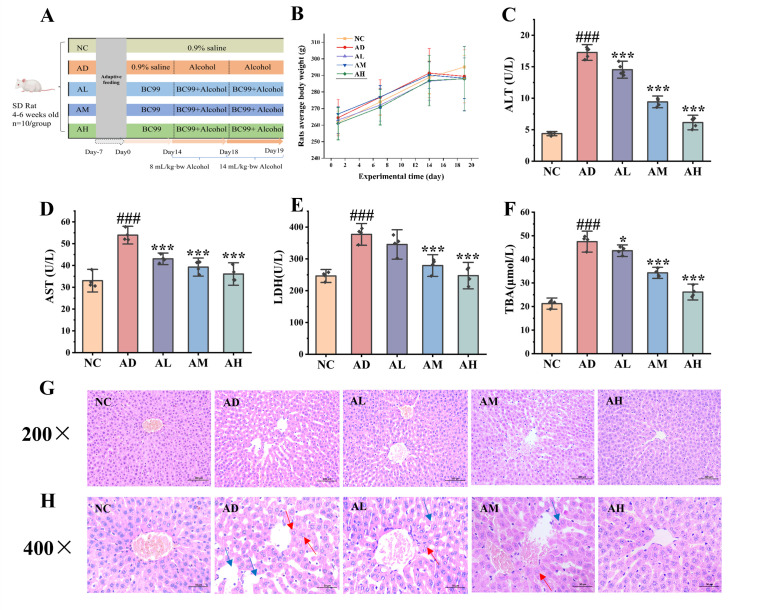
BC99 improved the liver injury of AALI rats. (**A**) Experiment design, (**B**) changes in body weight of rats, (**C**) plasma ALT level, (**D**) plasma AST level, (**E**) plasma LDH level, (**F**) plasma TBA level, (**G**) liver H&E staining, magnification 200×, (**H**) liver H&E staining, magnification 400×. The blue arrow represents disordered cell arrangement, while the red arrow represents cell necrosis. ### *p* < 0.001 vs. the NC group. * *p* < 0.05, *** *p* < 0.001 vs. the AD group.

After a one-week acclimatization period, both the model and control groups of rats were administered with saline via gavage at 9 a.m. daily, and three BC99 intervention groups were gavaged with 10^6^, 10^7^, and 10^8^ CFU/g·bw of BC99 bacterial solution according to the body weights of the rats every day for 19 days. Starting from the 15th day, the control group was gavaged with saline, and the model and BC99 groups were gavaged with white wine at a dose of 8 mL/kg·bw, and the dose of white wine was adjusted to 14 mL/kg·bw at the end of the last day of the experiment in order to cause an acute alcoholic liver injury model. The rats were fasted for 14 h, then sacrificed and blood, liver, ileum and cecum contents were collected. Four samples were randomly selected for the determination and analysis of relevant indicators (*n* = 4). The experimental design is shown in Figure 1A.

### 2.3. Liver Function Test

The blood samples were centrifuged at 4000 r/min and 4 °C for 15 min to obtain serum, which was subsequently used to measure the level of alanine aminotransferase (ALT), aspartate aminotransferase (AST), lactate dehydrogenase (LDH) and total bile acid (TBA). The assay reagent kits were procured from Nanjing Jiancheng Biotechnology Research Institute Co., Ltd. (Nanjing, China).

### 2.4. Histopathological Analysis

Hematoxylin–eosin (HE) staining was performed on liver tissue samples, which were fixed in 4% paraformaldehyde solution for 48 h, dehydrated, embedded in paraffin wax, sectioned, and observed under a microscope to assess histopathological changes.

### 2.5. Determination of Liver Alcohol Metabolism Enzymes

Liver tissues were homogenized in an ice water bath with nine volumes of physiological saline (4 °C, 12,000× *g*, 15 min). Take the supernatant and use reagent kits to measure the levels of alcohol dehydrogenase (ADH), acetaldehyde dehydrogenase (ALDH) and cytochromeP4502E1 (CYP2E1) in the liver. The ADH and ALDH test kit were purchased from BIOISCO (Lianyungang, China) Biotechnology Co., Ltd. The CYP2E1 ELISA kit was purchased from BYabscience (BYHS506035, Nanjing, China).

### 2.6. Measurement of Liver Oxidative Stress Indicators

Take a suitable quantity of liver tissue and combine with 9 times the amount of physiological saline and create a homogenate in an ice water bath (4 °C, 12,000× *g*, 15 min). Take the supernatant and use reagent kits to measure the levels of superoxide dismutase (SOD), glutathione (GSH) and malondialdehyde (MDA) in the liver. The reagent kits were purchased from Nanjing Jiancheng Biotechnology Research Institute Co., Ltd. (Nanjing, China).

### 2.7. Metabolomics Analysis

The samples (50 mg) were mixed with methanol and the supernatant was collected. After filtration by 0.22 µm filter prior, all metabolites were identified by mass spectrometry and their information was retrieved from the local database BiotreeDB (V3.0). Subsequently, the metabolite annotation information was re-checked and supplemented through the KEGG (http://www.genome.jp/kegg, accessed on 10 April 2024.) and HMDB (http://www.hmdb.ca, accessed on 10 April 2024.) databases. The annotation information of metabolites in the database was statistically analyzed. Combined with *t*-test, the differential metabolites were screened under the conditions of VIP > 1 and *p* < 0.05. The differential metabolite volcano map was analyzed by R(ggplot2) (3.3.5) software. Metabonomic analysis was completed on the BIOTREE biomedical technology Co., Ltd. (Shanghai, China).

### 2.8. qRT-PCR (Rat)

Total RNA was extracted from the liver using SteadyPure Universal RNA Extraction Kit (Accurate Bilogy, Changsha, China) following the manufacturer’s protocols, and measured by Multiskan (Multiskan FC, Thermo Fisher Scientific, Shanghai, China). The RNA was further reverse transcribed into cDNA (Evo M-MLV Mix Kit with gDNA Clean for qPCR, Accurate Bilogy, Changsha, China). SYBR Green Premix Pro Taq HS qPCR Kit from Accurate Biology Co., Ltd. (Changsha, China) was applied using quantitative real-time polymerase chain reaction (qRT-PCR, CFX96 Touch, Bio-Rad Laboratories Inc., Hercules, CA, USA). The amplification protocol involved an initial pre-amplification step at 95 °C for 30 s, followed by 40 cycles at 95 °C for 5 s and 60 °C for 30 s. The comparative Ct method (2^−ΔΔCt^) was utilized to calculate the levels of gene expression. All primers for these genes are shown in Table S1.

### 2.9. Experiment on C. elegans

#### 2.9.1. *C. elegans* Strains and Molding

Nematodes were cultured at a temperature of 20 °C in nematode growth medium (NGM) containing *E. coli* OP50. The strains utilized in this experiment comprise N2 (wild-type), GR2245(*skn-1*(*mg570*)), CF1028(*daf-16*(*mu86*)). The strains were preserved in the School of Food and Biotechnology, Henan University of Science and Technology.

Nematodes were lysed by hypochlorite solution to gain eggs, which were placed in NGMs of different strains for cultivation. Con and Mod group: *E. coli* OP50 suspension (1 × 10^7^ CFU/mL). BL group: BC99 suspension (1 × 10^7^ CFU/mL). BH group: BC99 suspension (1 × 10^8^ CFU/mL). After exposure for 48 h, transfer the *C. elegans* to a liquid medium with a 3% alcohol concentration for cultivation. After 24 h of exposure, the following parameters were measured. Each experiment was conducted three times, with no fewer than 30 nematodes measured in each experiment.

#### 2.9.2. Determination of Movement, Reproduction, and Longevity Indicators of *C. elegans*

Movement indicators: Select 10 *C. elegans* from each group and transfer them to blank NGM medium. Measure the frequency of head swings per minute and the number of body bends per 20 s.

Reproduction indicator: Select 10 *C. elegans* from each group and transfer them to blank NGM medium. Transfer them to new blank NGM medium every day. The sum of the offspring generations during the *C. elegans* detection period is the total egg production.

Life indicators: Refer to the method of Yun B et al. [20] and make modifications. The lifespan is calculated from egg growth until death.

#### 2.9.3. Determination of Antioxidant Capacity of *C. elegans*

H2DCF-DA was utilized as a fluorescent molecular probe for measuring the level of intracellular ROS in nematodes. After washing the nematodes with M9 buffer, they were transferred to centrifuge tubes. A total of 1 mL of 10 μmol/L H2DCFDA solution was added to each tube, and the tubes were incubated at 20 °C for 2 h with gentle mixing by inverting the tubes every 30 min. After the reaction, the nematodes were washed three times with M9 buffer. Afterward, they were anesthetized using 200 μL of 0.5 mmol/L levamisole and then placed on slides. Fluorescence microscopy was used for observation and imaging. The software Image J1.8.0 was utilized to analyze the fluorescence intensity of ROS. Count 10 nematodes per group.

After the *C. elegans* modeling was completed, the *C. elegans* were collected by centrifugation at 2500 r/min for 2 min and washed three times with 0.9% physiological saline. Centrifuge again to remove the supernatant, resuspend the insect solution in sterile water to 1mL, sonicate in an ice water bath, and take the supernatant (12,000 r/min, 10 min). Measure the contents of SOD, GSH, catalase (CAT), and MDA according to the method described in the kit. The reagent kits were procured from Nanjing Jiancheng Biotechnology Research Institute Co., Ltd. (Nanjing, China).

#### 2.9.4. qRT-PCR (*C. elegans*)

Reference Section 2.8, using β-actin as the internal reference gene. All primers for these genes are shown in Table S2.

#### 2.9.5. Mutant *C. elegans* Heat Stress Experiment

After modeling, each group randomly selected mutant GR2245 and CF1038 (*n* = 30) and placed them in a new plate. It was then placed in a 37 °C constant-temperature incubator and observed every two hours. The number of surviving nematodes was counted until all *C. elegans* died. The criteria for determining *C. elegans* death were the same as those used in the lifespan experiment.

### 2.10. Data Analysis

The experiment was plotted using Origin 2021 software; the experimental data were expressed as mean ± standard error and analyzed by one-way ANOVA or Kruskal–Wallis statistical analysis using SPSS25.0 software, with *p* < 0.05 indicating a significant difference, *p* < 0.01 indicating a highly significant difference, and *p* < 0.001 indicating an extremely highly significant difference.

## 3. Results

### 3.1. BC99 Improved Liver Function in AALI Rats

Changes in the body weight of rats during the experiment are shown in Figure 1B. Body weight gradually decreased following alcohol administration via gavage, though the differences were not statistically significant. Liver function index results are presented in Figure 1C–F. The levels of ALT, AST, LDH, and TBA were significantly elevated in the AD group compared to the NC group (*p* < 0.001), indicating severe liver damage caused by alcohol consumption. In contrast, these markers were significantly reduced in the BC99-treated groups (*p* < 0.05), with reductions corresponding to the intervention dose. Specifically, compared to the AD group, ALT levels in the AH group decreased by 64.45% (*p* < 0.001), AST by 33.09% (*p* < 0.001), LDH by 34.4% (*p* < 0.001), and TBA by 44.98% (*p* < 0.001).

### 3.2. BC99 Alleviated Liver Pathological Injury in AALI Rats

The protective effect of BC99 on AALI in rats was further confirmed through HE staining of liver tissue. Liver histopathology at different magnifications is shown in Figure 1G,H. Hepatocytes in the NC group exhibited an orderly arrangement with distinct outlines, no signs of degeneration or necrosis, and large, round nuclei. In contrast, liver tissue in the AD group displayed significant disorganization, shrunken hepatocytes, cloudy and swollen stromal cells, extensive necrosis, and reduced cytoplasmic density, consistent with alcohol-induced liver injury. In the BC99-treated groups, liver damage was visibly mitigated, showing reduced necrosis, orderly hepatic cord arrangement, and hepatocytes radiating from the central vein. Notably, the liver morphology in the AH group closely resembled that of the NC group.

### 3.3. BC99 Regulated the Activity of Alcohol Metabolism Enzymes in AALI Rats

**Figure 2:**
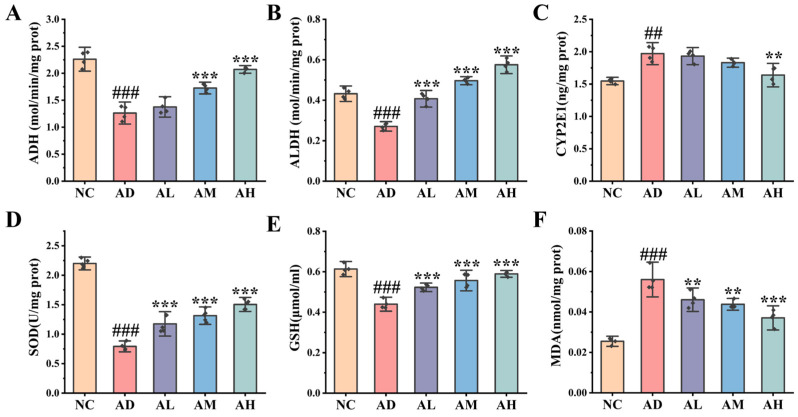
BC99 reduced liver oxidative stress injury in AALI rats. (**A**) Liver ADH activity. (**B**) Liver ALDH activity. (**C**) Liver CYP2E1 level. (**D**) Liver SOD activity. (**E**) Liver GSH content. (**F**) Liver MDA content. ## *p* < 0.01, ### *p* < 0.001 vs. the NC group. ** *p* < 0.01, *** *p* < 0.001 vs. the AD group.

As can be seen from Figure 2A–C, the activities of ADH and ALDH in the liver of rats in the AD group were significantly decreased, while the activity of CYP2E1 was significantly increased (*p* < 0.001), but this situation was improved in three BC99 groups, among which the ADH activity and ALDH activity in the AH group were increased by 64.07% and 112.52%, respectively (*p* < 0.001). At the same time, among the three BC99 groups, only the activity of CYP2E1 in the high-dose group was significantly different from that in the AD group (*p* < 0.01), and compared with the AD group, the activity of CYP2E1 decreased by 16.84%.

### 3.4. BC99 Reduced Oxidative Stress Levels in the Liver of AALI Rats

We further studied the effect of BC99 on oxidative stress in AALI rats’ liver (Figure 2D–F). Overall, compared with NC group, the levels of SOD and GSH in the liver of rats in AD group were significantly decreased (*p* < 0.001). Compared with the AD group, the levels of SOD and GSH in the liver of the AH group increased by 89.86% and 36.14%. Moreover, in comparison to the NC group, the concentration of MDA in the AD group exhibited a markedly elevated level (*p* < 0.001). Compared with the AD group, the MDA level in the AH group decreased by 33.75% (*p* < 0.001), and this decrease was found to be directly associated with the dosage. In summary, BC99 supplementation successfully reduced the level of oxidative stress in the liver, but it was dose-related.

### 3.5. BC99 Alleviated Alcohol-Induced Liver Dysregulated Metabolic Profiles

In order to further understand the protective mechanism of *W. coagulans* BC99 in improving acute alcoholic liver injury, we used the non-targeted metabonomics method based on LC-MS to extract and detect the liver metabolites of mice.

**Figure 3:**
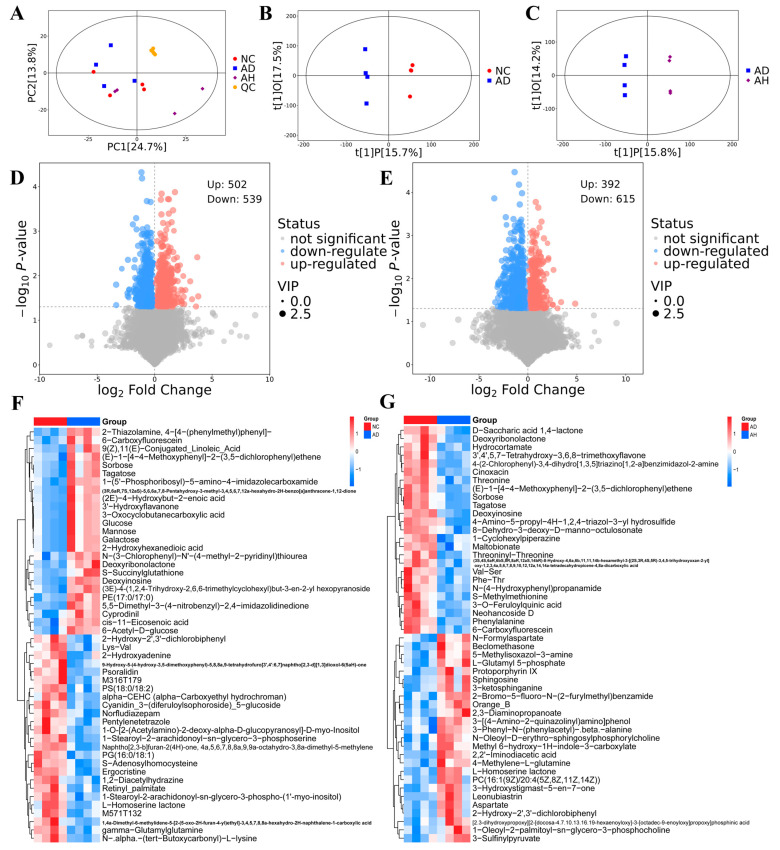
BC99 altered liver metabolites in AALI rats. (**A**) PCA analysis. (**B**) OPLS-DA score scatter plot (NC vs. AD). (**C**) OPLS-DA score scatter plot (AD vs. AH). (**D**) Volcanic diagram of differential metabolites (NC vs. AD). (**E**) Volcanic diagram of differential metabolites (AD vs. AH). (**F**) Heat map of the top 50 differential metabolites (NC vs. AD). (**G**) Heat map of the top 50 differential metabolites (AD vs. AH).

Through PCA analysis of the samples, we can obtain a preliminary understanding of the overall situation of the differences of metabolites among the groups, and this can also reflect the variation within each group. The results of PCA showed that the samples of the NC group and AD group partially overlapped, which may be related to the acute alcoholism model, and the AD group and AH group were clearly distinguished (Figure 3A). In order to explore the essence of the differences among different groups, supervised orthogonal partial least squares discriminant analysis (OPLS-DA) was performed on the NC group, AD group and AH group, respectively. According to the OPLS-DA score chart, the difference between the NCvsAD group and the ADvsAH group was obvious, which indicated that BC99 significantly changed the composition of liver metabolites in AALI rats, and the composition was similar to that in the NC group (Figure 3B,C). According to VIP > 1 and *p* < 0.05, the potential biomarkers were screened from the online database. The analysis of the differential metabolite set showed that among the 1041 biomarkers screened by the control group and the model group, 502 biomarkers were upregulated and 539 biomarkers were downregulated. A total of 1007 biomarkers were screened from the model group and the high-dose group. Compared with the model group, BC99 intervention resulted in 392 biomarkers being upregulated and 615 biomarkers being downregulated (Figure 3D,E). Both alcohol and BC99 interventions can cause significant changes in metabolites in the liver (Figure 3F,G).

**Figure 4:**
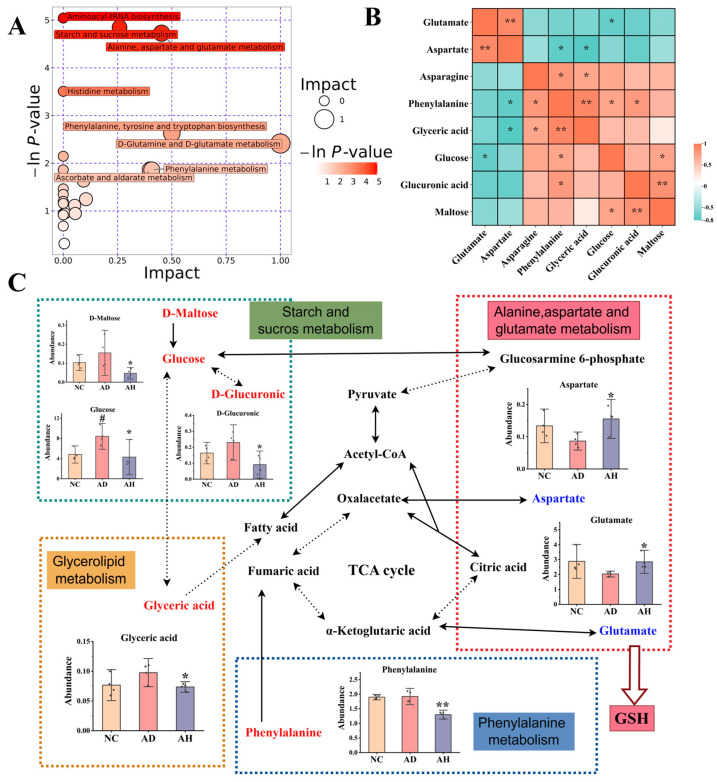
Main biomarkers and metabolic networks of BC99 intervening AALI (AD vs. AH). (**A**) Metabolic pathway analysis of differential metabolites. Each bubble represents a metabolic pathway. The larger the bubble, the more important the pathway. (**B**) Correlation analysis of differential metabolites, * *p* < 0.05, ** *p* < 0.01. (**C**) The network of key metabolic pathways. Different colors indicate metabolites that were upregulated (blue) or downregulated (red) in the AH group compared with the AD group. Solid arrow indicates single process. Dotted arrow indicates multiple processes. Bar graphs show the levels of important differential metabolites, # *p* < 0.05 vs. the NC group. * *p* < 0.05, ** *p* < 0.01 vs. the AD group.

In order to better understand biomarkers, we focused on the analysis of differential metabolites between the AD group and AH group. We conducted enrichment analysis and topological analysis to identify the key pathway with the highest correlation with metabolite differences. The results showed that BC99 improved the dysregulated metabolic profiles induced by alcohol through various metabolic pathways (Figure 4A). Among them, seven pathways with influencing factors > 0.1 are alanine, aspartate and glutamate metabolism, Starch and sucrose metabolism, Phenylalanine, tyrosine and tryptophan biosynthesis, D-Glutamine and D-glutamate metabolism, Ascorbate and aldarate metabolism, Phenylalanine metabolism, Glycerolipid metabolism. These seven metabolic pathways involve eight different metabolites, including Aspartate, Glutamate, L-Asparagine, Glucose, D-Maltose, D-Glucuronic, Phenylalanine, Glyceric acid. Subsequently, we analyzed the interactions of relevant metabolites and integrated their main metabolic networks (Figure 4B,C). Compared with the AD group, the contents of L-asparagine, glucose, D-maltose, D-glucoronic, phenyllalanine and glycoacid in the AH group decreased significantly, while the contents of aspartate, glutamate increased significantly.

### 3.6. BC99 Upregulated Antioxidant-Related Gene Levels

**Figure 5:**
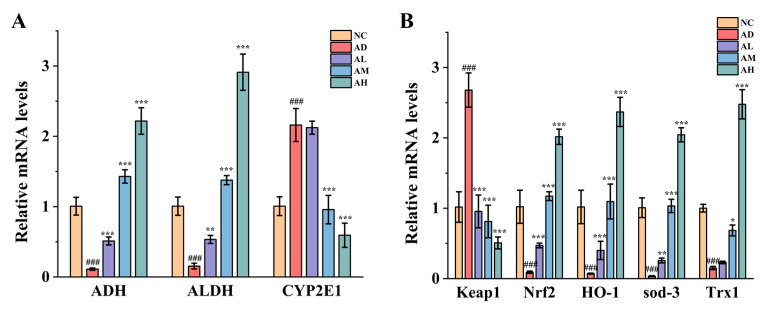
BC99 enhanced the expression of antioxidant-related genes in AALI rats. (**A**) Expression of genes related to alcohol metabolism. (**B**) Expression of genes related to Nrf2 pathway. ### *p* < 0.001 vs. the NC group. * *p* < 0.05, ** *p* < 0.01, *** *p* < 0.001 vs. the AD group.

We further explored the gene level related to oxidative stress in liver (Figure 5A,B). It was found that alcohol intake decreased *ADH* level and increased *CYP2E1* level, suggesting that after a large amount of alcohol intake, alcohol was mainly carried out through the CYP2E1 pathway, thus producing a large amount of reactive oxygen species and causing oxidative stress damage to the liver. In addition, alcohol significantly inhibited the expression of *Nrf2*, *HO-1*, *Keap1*, *sod-3* and *Trx1* in the liver, which eventually led to liver injury. The BC99 intervention group significantly inhibited the expression of *CYP2E1* gene, and increased the expression of *Nrf2*, *HO-1*, *Keap1*, *sod-3* and *Trx1* gene, among which the high-dose group had the best effect. The above results indicate that the BC99 intervention group can improve the oxidative stress by regulating the expression of the Nrf2 pathway and *CYP2E1* gene.

### 3.7. Spearman Correlation Analysis of Differential Metabolites

**Figure 6:**
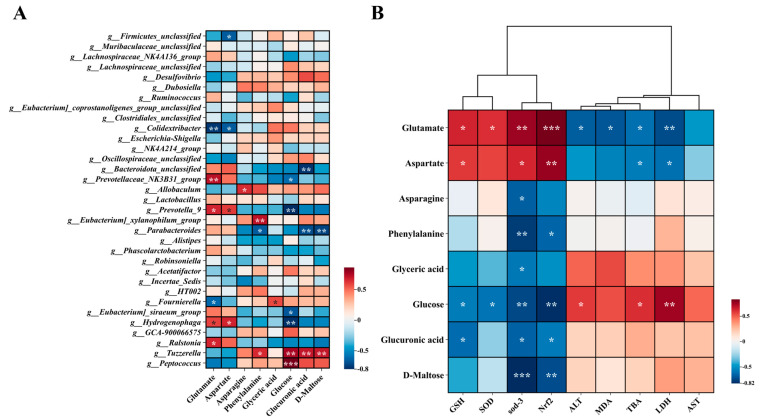
Spearman correlation analysis of differential metabolites. (**A**) Spearman correlation analysis between liver differential metabolites and intestinal flora. (**B**) Spearman correlation analysis of differential metabolites and phenotypes. * *p* < 0.05, ** *p* < 0.01, *** *p* < 0.001.

Our laboratory obtained the data of 16S intestinal flora in the rat model of acute alcoholic liver injury in the early stage [21], and analyzed its correlation with liver metabolites in this study to better explain the changes in differential metabolites (Figure 6A). Correlation analysis showed that Glutamate had a significant positive correlation with *Prevotellaceae_NK3B31_group*, *Ralstonia* and *Hydrogenophaga* in the AH group, and a significant negative correlation with *Fournierella* in the AD group. Glucose was negatively correlated with the different bacteria genera *Prevotellaceae_NK3B31_group* and *Hydrogenophaga* in the AH group. There was a significant negative correlation between Phenylalanine and *Parabacteroides*, a different bacterium in the AH group. Glyceric acid was positively correlated with *Fournierella*, a different strain in the AD group. Studies have proved that the changes of these differential metabolites may be related to the differences of intestinal flora.

In addition, we also analyzed the correlation between differential metabolites and oxidative stress index and liver injury index (Figure 6B). It was found that Glutamate was positively correlated with antioxidant genes, SOD and GSH, and negatively correlated with ALT, TBA and LDH. Aspartate was positively correlated with antioxidant genes and GSH, and negatively correlated with TBA and LDH. However, Glucose was negatively correlated with antioxidant genes, SOD and GSH, and positively correlated with ALT, TBA and LDH. This result shows that alcoholic liver injury may be related to the increase in glucose. The changes of two amino acids may be the reason for reducing oxidative stress and alleviating liver injury.

### 3.8. BC99 Alleviated Alcohol-Induced Damage to C. elegans Through the Nrf2/SKN-1 Pathway

With its rapid reproduction, short lifespan, cost-effective bioassays, and similarities to human biological processes, *C. elegans* has been extensively employed for toxicological assessment [22]. To further elucidate the mechanism by which BC99 mitigates alcohol-induced oxidative stress, nematodes were chosen as the model for further investigation.

#### 3.8.1. BC99 Enhanced the Life Function of Alcohol-Induced N2 Nematodes

The exercise index, which reflects basic nervous system functionality, was used to evaluate the protective effects of BC99 against alcohol-induced oxidative stress in *C. elegans*. Compared to the Con group, the Mod group exhibited a significant reduction in head-swing frequency and body-bending counts (*p* < 0.05). Following BC99 intervention, both parameters were significantly improved (Figure S1A,B). Similarly, reproductive capacity was assessed, revealing that the BH group showed a significant increase in egg laying compared to the Mod group (Figure S1C).

Lifespan analysis demonstrated that the survival curve of the Mod group shifted to the left, with an average lifespan reduced by 9.5% compared to the Con group. In contrast, BC99-treated groups exhibited survival curves shifted to the right, with average lifespans in the BL and BH groups extended by 25.19% and 38.87%, respectively (Figure S1D,E). These findings suggest that BC99 effectively mitigates alcohol-induced damage in *C. elegans*.

#### 3.8.2. BC99 Reduced Alcohol-Induced Oxidative Stress in N2 Nematodes

**Figure 7:**
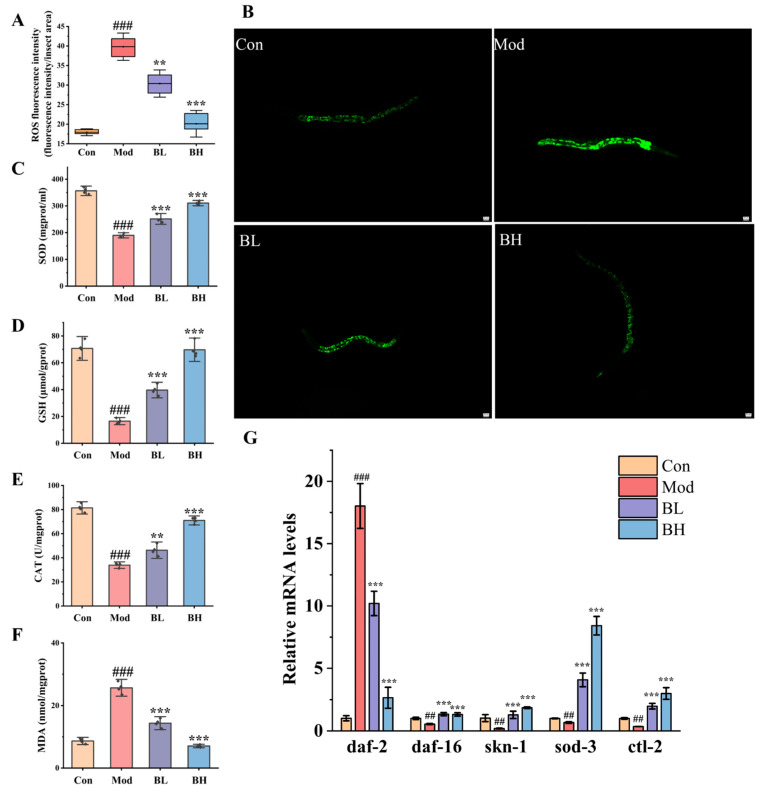
BC99 enhanced the levels of antioxidant-related genes and enzymes in N2 nematodes. (**A**) Relative fluorescence intensity of ROS in N2 nematodes. (**B**) Typical images of ROS in N2 nematodes (scale bar 20 μm). (**C**) SOD activity. (**D**) GSH content. (**E**) CAT activity. (**F**) MDA content. (**G**) Relative gene expression of antioxidant related genes. ## *p* < 0.01, ### *p* < 0.001 vs. the Con group. ** *p* < 0.01, *** *p* < 0.001 vs. the Mod group.

We utilized H2DCF-DA probes to measure the levels of ROS. The ROS levels in the Mod group showed a significant increase compared to the Con group (*p* < 0.001). Compared with the Mod group, the BC99 intervention significantly reduced ROS levels, while the BH group decreased by 49.51% (Figure 7A,B). At the same time, the results showed that the levels of SOD, CAT and GSH in nematodes were significantly decreased and MDA was significantly increased (*p* < 0.001). After BC99 intervention, the levels of SOD, CAT, GSH and MDA in the BH group have recovered to the level of the Con group (Figure 7C–F). The above results show that BC99 can alleviate the oxidative stress damage caused by alcohol, so as to achieve the effect of alleviating alcohol damage.

#### 3.8.3. BC99 Influenced Nematodes by Regulating Nrf2/SKN-1 Pathway

Furthermore, the expression of antioxidant-related genes in N2 nematodes was detected. As shown in Figure 7G, the expression level of *skn-1* in Mod nematodes was significantly lower than that in the control group, but the *skn-1* gene was significantly upregulated after treatment with BC99. Similarly, *daf-16* and its downstream target genes *sod-3* and *ctl-2* showed a reduction in expression in the Mod group. BC99 upregulated the expression of these genes. *daf-2* and *daf-16* are negatively regulated. Alcohol increased the expression level of *daf-2* in Mod, while BC99 decreased the expression level of *daf-2*. In combination, these findings indicated that *daf-16* and *skn-1* play a role in BC99′s resistance to oxidative stress.

#### 3.8.4. The Effect of BC99 on Oxidative Stress in the Mutant Strain Was Significantly Weakened

**Figure 8:**
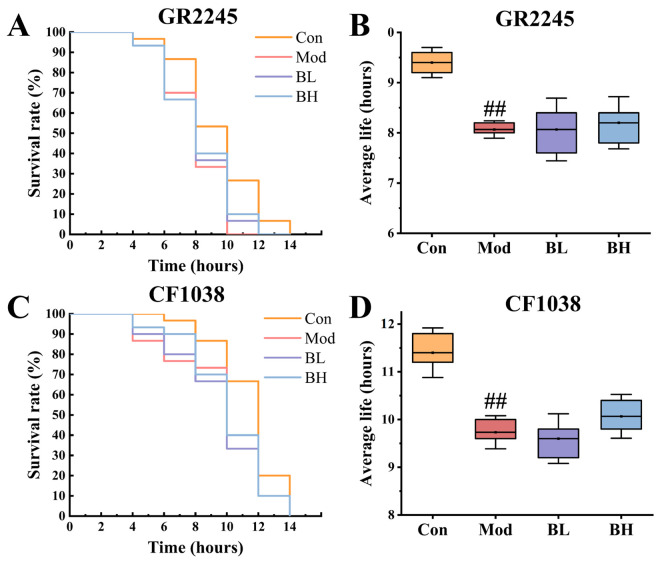
BC99 significantly attenuated the antioxidant stress resistance of GR2245 and CF1038 mutants. (**A**) GR2245 mutant survival curve. (**B**) GR2245 mutant average life. (**C**) CF1038 mutant survival curve. (**D**) CF1038 mutant average life. ## *p* < 0.01 vs. the Con group.

Under normal conditions, the content of active oxygen in nematodes is in a dynamic equilibrium state. The high temperature of 37 °C will cause dysregulated metabolic profiles in nematodes and produce a large amount of active oxygen, which will lead to oxidative stress and losses. Therefore, the ability to resist thermal stress has become an important indicator of the antioxidant activity of nematodes [23]. In order to confirm the importance of sskn-1, daf-16 and its downstream targets in BC99′s resistance to alcohol-induced oxidative stress, we conducted stress resistance experiments using CF1038 (a *daf-16*(-) mutant) and GR2245 (a *skn-1*(-) mutant) (Figure 8A–D). The findings indicated that there was no significant disparity in the average lifespan of the two mutant strains between the BC99 group and Mod group. BC99 had lost its ability to improve the stress resistance of alcohol-induced nematodes. These results provided further validation for our previous conclusions.

## 4. Discussion

Alcohol-mediated oxidative liver injury is assuredly related to the overproduction of ROS and the presence of oxidative stress in the liver [24]. Developing effective antioxidants capable of neutralizing free radicals and mitigating oxidative damage is a promising strategy for preventing acute alcoholic liver injury AALI. Probiotics, particularly BC99, show great potential in addressing alcoholic liver injury due to their unique probiotic properties [25,26]. This study expands our understanding of the physiological effects of *W. coagulans* BC99.

Excessive alcohol intake causes significant alterations in liver function. Alcohol can damage hepatocytes, resulting in the release of enzymes such as AST, ALT, LDH, and TBA, which in turn leads to elevated serum levels of these markers [27]. Our results demonstrated that alcohol exposure significantly increased serum AST, ALT, LDH, and TBA levels, indicating severe liver damage. Intervention with BC99 significantly improved these liver function markers in a dose-dependent manner. Histopathological evaluation through HE staining further confirmed that BC99 alleviated alcohol-induced liver pathological changes, underscoring its protective effect on acute alcohol-induced liver injury.

Oxidative stress is the key factor in the pathogenesis of ALD [28]. The present study also confirmed this observation. A small amount of alcohol enters the body and is metabolized by the alcohol dehydrogenase system in the liver. When drinking a lot at one time, the SYP2E1 microsomal alcohol oxidation system will be activated, which will produce a large number of ROS, which is the key to stimulating the body’s oxidative stress response [29], so the level of CYP2E1 can reflect the body’s oxidative stress level. GSH is the reducing substrate of GSH-Px in the process of scavenging hydrogen peroxide, and it can also directly scavenge peroxide free radicals [30]. MDA is the final product of lipid peroxidation [31]. Combined with antioxidant enzyme SOD, the above indexes can comprehensively evaluate the level of oxidative stress in the liver. The results showed that alcohol significantly decreased the levels of SOD and GSH and increased the level of MDA, while intake of BC99 improved the above antioxidant parameters, inhibited the decline of antioxidant enzymes and GSH, and decreased the level of MDA. Fang [32] showed that *Lactobacillus plantarum* CMU995 could also inhibit the decrease in SOD, GSH-Px and GSH in mice liver caused by alcohol, and increase the content of MDA. At the same time, intake of BC99 reduces the level of CYP2E1 and increases the level of ADH, thus reducing the ROS produced by alcohol metabolism to protect the liver, which is the same as the conclusion reached by Jiang et al. [33].

Metabolomic analysis revealed that alcohol disrupts key metabolic pathways, including alanine, aspartate, and glutamate metabolism, which are closely linked to alcohol dependence [34]. We found that compared with the AD group, the glutamate level in the AH group increased significantly. Glutamate comes from many sources, including endogenous protein catabolism, dietary intake of protein and monosodium glutamate, and digestive free L-glutamic acid, which comes from the degradation of cavity peptide [35]. L-glutamic acid, L-cysteine and glycine have the capability to produce GSH, which serves as the primary low-molecular-weight antioxidant in cells. The increase in its content may help protect cells from oxidative damage [36], which is consistent with the determination of GSH’s upward trend. Aspartic acid is a non-essential amino acid, which is the precursor of amino acids such as threonine, lysine, methionine and isoleucine. The decrease in aspartic acid level may be related to alcohol-induced ketosis [37]. Hinton et al. pointed out that aspartic acid can be used as a potential biomarker to treat alcohol-dependent diseases [38]. In addition, Glu and Asp are important neurotransmitters in the central nervous system, mainly involved in the detoxification reaction of intracellular oxygen free radicals, and can regulate the functions of nervous system, reproduction, memory and exercise [39]. The correlation analysis of differential metabolites showed that glutamic acid and aspartic acid may also have a regulatory relationship with antioxidant pathways, but further research is needed. Phenylalanine is one of the essential amino acids in the human body, and there are two metabolic pathways in the body [40]. One is to produce tyrosine and then metabolize it into neurotransmitters such as dopamine. Another way is to produce phenylpyruvate. In addition, phenylalanine can induce oxidative stress [41], and in a pathological state, phenylalanine can also produce harmful substances to damage the body [42], which may be the reason why BC99 significantly reduces phenylalanine in AALI rats. The tricarboxylic acid (TCA) cycle, a central pathway for carbohydrate and lipid metabolism, was disrupted in AALI rats, as evidenced by altered levels of TCA intermediates like maltose, glucose, and glyceric acid. Excess glucose exacerbates oxidative stress through pathways such as glycolysis and the pentose phosphate pathway [43,44,45], causing oxidative stress damage in the body, while BC99 could significantly reduce the glucose level. These data further indicate that the alleviation of acute alcoholic liver injury by BC99 is closely related to antioxidant activity. Combined with the results of enzyme activity determination, we speculate that BC99 may eliminate active oxygen through a fine defense system composed of antioxidant-related enzymes such as SOD and non-enzymatic antioxidants such as glutathione, thus reducing oxidative stress, thus alleviating acute alcoholic liver injury.

*C. elegans*’ nervous system, reproductive system and digestive system are simple and perfect in structure, highly homologous to human beings, and have great advantages in toxicology research, and are widely used in toxicity evaluation and mechanism research [46]. Previous laboratory studies indicated that BC99 exerts dose-dependent protective effects in *C. elegans* [47]. We selected the dose with a better intervention effect to study the alleviation of oxidative stress in drunken nematodes by BC99. This study found that nematodes exposed to alcohol showed dyskinesia, decreased longevity and reproductive capacity, which may be related to alcohol promoting ROS production and inducing oxidative stress [48,49]. Numerous studies have demonstrated that the stimulation of the insulin IGF-1 signal (IIS pathway) (which is highly preserved across nematodes and humans) confers resistance to oxidative stress [50]. Nrf2 participates in the regulation of the antioxidant defense system and various antioxidant mechanisms in vivo. Nrf2 can also activate the expression of many downstream antioxidant factors and cytoprotective genes, such as HO-1, sod-3 and Trx1, which work together to remove free radicals and alleviate oxidative stress. DAF-16 and SKN-1 (homologues of mammalian Nrf2 transcription factor) are involved in the insulin/insulin-like growth factor-1 signaling pathway of *C. elegans*, and are crucial for enhancing stress resistance [51]. In addition, *daf-16* can induce the expression of various antioxidant genes to resist oxidative stress, such as *sod-3/5* and *ctl-1/2* [52].

Our results demonstrated that alcohol exposure suppressed the expression of *skn-1*, *daf-16*, and their downstream targets, thereby impairing antioxidant enzyme activity and exacerbating oxidative stress. BC99 treatment upregulated the expression of *skn-1*, *daf-16*, *sod-3*, and *ctl-2*, enhancing the host’s antioxidant defense system and restoring enzyme activity. Previous studies have shown that probiotics can also alleviate oxidative stress through alternative pathways. For instance, Zhang et al. [53] reported that Probio-M9 regulates *skn-1* via the *daf-2* signaling pathway, independent of *daf-16*. Similarly, *Lactobacillus maydis* LB1 induces the production of antimicrobial peptides and defense molecules (such as LYS-7 and CLEC-85) through p38MAPK and IIS, which enhances the resistance of *C. elegans* [54]. Finally, the reduced efficacy of BC99 in SKN-1 and DAF-16 mutant strains confirms the critical roles of these pathways in BC99-mediated antioxidant activity. These findings highlight BC99′s potential as a therapeutic agent for alleviating alcohol-induced oxidative stress through the regulation of key antioxidant signaling pathways.

## 5. Conclusions

Excessive alcohol intake will cause oxidative damage and liver dysregulated metabolic profiles, resulting in acute alcoholic liver injury, while *W. coagulans* BC99 restored liver dysregulated metabolic profiles and activated the Nrf2/SKN-1 pathway. On the one hand, *W. coagulans* BC99 can improve oxidative stress by regulating the levels of amino acids such as glutamic acid and aspartic acid and improving the disorder of glucose metabolism, which leads to a significant increase in the level of non-enzymatic antioxidants. On the other hand, *W. coagulans* BC99 enhances the transcription and function of antioxidant-related enzymes by elevating the levels of *daf-16*, *Nrf2*/*skn-1* and their subsequent antioxidant genes. *W. coagulans* BC99 can eliminate ROS and anti-oxidative stress through the fine defense system of antioxidant enzymes and non-enzymatic antioxidants, thus reducing the damage caused by alcohol. This study provides research ideas and a theoretical basis for *W. coagulans* BC99 to alleviate alcohol-induced oxidative liver injury.

We believe that this article still has some limitations. Firstly, the conclusions from the rat experiments have not been fully validated in *C. elegans*. BC99 regulated the levels of alcohol metabolizing enzymes, especially reducing the level of CYP2E1, but the specific regulatory mechanism still needs further investigation. In addition, this study used two models to explore the impact of BC99 on alcohol-induced oxidative stress. However, human alcohol damage factors are more complex, and further clinical trials are needed to investigate the conclusion that BC99 alleviates alcohol-induced oxidative stress.

## Data Availability

The original contributions presented in the study are included in the article; further inquiries can be directed to the corresponding authors.

## Supplementary Materials

The following supporting information can be downloaded at: https://www.mdpi.com/article/10.3390/antiox14010117/s1, Table S1: Rat gene sequence; Table S2: *Caenorhabditis elegans* gene sequence; Figure S1: BC99 enhanced the life function of alcohol induced N2 nematodes. (A) Head swing frequency. (B) Number of body bends. (C) Fecundity. (D) N2 nematode survival curve. (E) N2 nematode average life. # *p* < 0.05, ## *p* < 0.01, ### *p* < 0.001 vs. the Con group. * *p* < 0.05, ** *p* < 0.01, *** *p* < 0.001 vs. the Mod group.
