# Supplementary material for: Weizmannia coagulans BC99 Attenuates Oxidative Stress Induced by Acute Alcoholic Liver Injury via Nrf2/SKN-1 Pathway and Liver Metabolism Regulation
Source: Antioxidants (Basel). 2025 Jan 20;14(1):117. doi: 10.3390/antiox14010117 (PMC11760874; doi:10.3390/antiox14010117)
Supplement: Supplementary file 1 [file antioxidants-14-00117-s001.zip › antioxidants-3398393-supplementary.pdf]

Table S1. Rat gene sequence.

Gene	Primer sequence
Nrf2-F	TGCCTTCCTCTGCTGCCATTAG
Nrf2-R	CCGTGCCTTCAGTGTGCTTC
HO-1-F	GAACTTTCAGAAGGGTCAGGTGTC
HO-1-R	CTGCTTGTTTCGCTCTATCTCCTC
sod-3-F	ATCACAGGCTTGGTCCTCTTCC
sod-3-R	TGGCGTGGTTGGAGGTGTTC
ADH1C-F	GTTGGTCTGTCTGTCGTCATTGG
ADH1C-R	CAGTCAGTGGCACCTAACTCTTTG
Aldh3a1-F	GCTGGAGAAGGCTGTGTAGGAG
Aldh3a1-R	CAACTGCTGGATTCGGAACTGC
Cyp2e1-F	TTGTTTCTGCTCCTGTCTGCTATTC
Cyp2e1-R	GGGATACTGCCAAAGCCAACTG
Keap1-F	TGCTCAACCGCTTGCTGTATG
Keap1-R	GTAATCATCCGCCACTCATTCCTC
trx1-F	AAGGAAGCTTTTCAGGAGGC
trx1-R	GGCAGTCATCCACGTCTACT
β-actin-F	CACGATGGAGGGGCCGGACTCATC
β-actin-R	TAAAGACCTCTATGCCAACACAGT

Table S2. *Caenorhabditis elegans* gene sequence.

Gene	Primer sequence
sod-3-F	CCAACCAGCGCTGAAATTCAATGG
sod-3-R	GGAACCGAAGTCGCGCTTAATAGT
ctl-2-F	GAGAATGTGCCAGAACTTTGC
ctl-2-R	CTTGACACGAGCTCCAAAATC
daf-16-F	CTTCAAGCCAATGCCACTACC
daf-16-R	GGAGATGAGTTGGATGTTGATAGC
skn-1-F	GACGTCAATTTATGGAGTGTCG
skn-1-R	GAAGATGTTTTGTCGTGATCCG
daf-2-F	ATGAAAGCGAAGCAGCGAGAAGG
daf-2-R	CGTCCGAACTTCCGCATCACTC
β-actin-F	TCGGTATGGGACAGAAGGAC
β-actin-R	CATCCCAGTTGGTGACGATA

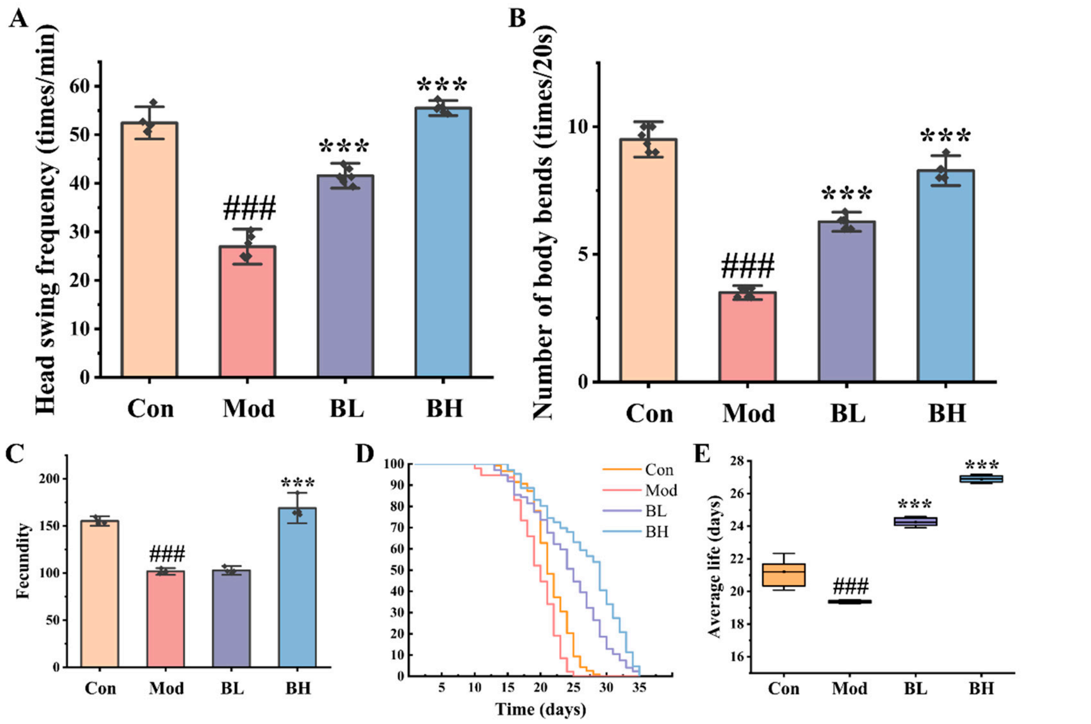

Figure S1. BC99 enhanced the life function of alcohol induced N2 nematodes. (A) Head swing frequency. (B) Number of body bends. (C) Fecundity. (D) N2 nematode survival curve. (E) N2 nematode average life. ### $p < 0.001$ vs. the Con group. *** $p < 0.001$ vs. the Mod group.
